# With Daffodil and Dragon at Walthamstow

**Published:** 1913-01-04

**Authors:** 


### WITH DAFFODIL AND DRAGON AT WALTHAMSTOW.

The spirit of Christmas at the Walthamstow Hospital was certainly shown by the smiling faces and happy disposition of one and all. The men's ward was decorated to represent Japan. The five doorways were arched with the national flower, and with a display of lanterns, dolls, scrolls, and real chrysanthemums. A most pleasing effect was produced. "Ye Olde English Xmas" was the note which the women's ward preferred to strike, chains of holly being hung from side to side, a Christmas tree taking a centre position under the guardianship of a life-size model of Father Christmas, and anyone who stood in the ward could imagine as he looked at the icicle-hung windows, upon which an excellent snow effect had been obtained, that a real old-fashioned Christmas was with us once again.

The two children's wards had taken "Wales" for their decoration scheme, and at every turn the eye was met with daffodil and dragon, not forgetting to mention a magnificent tree which occupied one end of the ward. Christmas Day was spent much as every Christmas Day is spent in hospitals. There was first the excitement of the Christmas stockings filled with the generous gifts of many—indeed, the staff believe that perhaps never have so many gifts been received before as were received this year. Then there was the Christmas dinner, and in the afternoon every patient had two visitors who stayed to tea, and when at last the time came for their departure the patients were entertained by a gramophone, the generous gift of the house surgeon. The Christmas festivities were continued on Saturday, when a large number of children who had been in-patients during the year were entertained in the out-patient department to tea and a Christmas tree. Every child received two gifts and with a parting gift of an orange went home to dream of that wonderful personage who still finds a place in the child's mind, the ever-green "Father Christmas."

